# Apoplexy

**Published:** 1893-11

**Authors:** 


### APOPLEXY.

Apoplexy in the beginning, Aconite (with hard and quick pulse); Opium (with full and slow pulse); Lachesis (with weak and rapid pulse). Of course these are not the only remedies for apoplexy.

